# Polyester Sheet Plastination: Technical Foundations, Methodological Advances, Anatomical Applications, and AQUA-Based Quality Analysis

**DOI:** 10.3390/polym17233177

**Published:** 2025-11-29

**Authors:** Nicolás E. Ottone, Carlos Torres-Villar, Ricardo Gómez-Barril, Josefa Baeza-Fernández, Víctor Hugo Rodríguez-Torrez, Carlos Veuthey

**Affiliations:** 1 Laboratory of Plastination and Anatomical Techniques, Universidad de La Frontera, Temuco 4780000, Chile; carlos.veuthey@ufrontera.cl; 2 Adults Integral Dentistry Department, Center for Research in Dental Sciences (CICO), Faculty of Dentistry, Universidad de La Frontera, Temuco 4780000, Chile; 3 Center of Excellence in Morphological and Surgical Studies (CEMyQ), Universidad de La Frontera, Temuco 4780000, Chile; 4 Doctoral Program in Morphological Sciences, Universidad de La Frontera, Temuco 4780000, Chile; carlos.torres@uss.cl; 5 Departamento de Ciencias Morfológicas, Facultad de Ciencias, Universidad San Sebastián, Puerto Montt 5501842, Chile; 6 Faculty of Dentistry, Universidad de La Frontera, Temuco 4780000, Chile; r.gomez06@ufromail.cl (R.G.-B.); j.baeza02@ufromail.cl (J.B.-F.); 7 Medicine and Dentistry Department, Universidad Privada del Valle, La Paz 5911, Bolivia; vrodriguezt@univalle.edu; 8 Postgraduate and Research Direction, Faculty of Dentistry, Universidad de La Frontera, Temuco 4780000, Chile

**Keywords:** sheet plastination, polyester resin, anatomical education, anatomical preservation, AQUA assessment

## Abstract

**Background/Objectives:** Plastination with polyester resin is a consolidated technique for anatomical preservation, particularly valuable in neuroanatomy education and radiological correlation. This review synthesizes the principles, technical evolution, methodological variations, applications, and limitations of polyester-based sheet plastination methods (P35, P40, P45). **Methods:** Key documents were analyzed to trace the transition from P35, recognized for excellent gray-white matter contrast but technical complexity, to P40, offering greater transparency, lower viscosity, improved strength, and simplified UV-curing. P45 was also reviewed, especially for large body sections using water-bath curing. Innovations included vertical curing chambers, active-passive vacuum cycles, resin reformulations, and strategies to reduce tissue shrinkage. Methodological quality was assessed with the AQUA tool, which evaluates five domains: Objectives, Study Design, Methodology, Descriptive Anatomy, and Results Reporting. **Results:** Plastination proved applicable in medical and veterinary education, as well as morphometric and imaging-based research, improving anatomical understanding and CT/MRI correlation. AQUA analysis revealed low risk of bias in Objectives and Descriptive Anatomy, but frequent unclear or high-risk assessments in Study Design, Methodology, and Results Reporting, mainly due to limited details on sample selection, resin handling, curing, and reproducibility. Publications after 2010 showed improved methodological rigor, reflecting growing standardization and better reporting. **Conclusions:** Polyester sheet plastination remains a versatile, high-impact tool, though it requires specialized infrastructure, trained personnel, and strict environmental control. Future development should focus on protocol standardization, international dissemination, integration with digital technologies (3D models, virtual reality), and sustainable alternatives. Progress depends on inter-institutional collaboration, technical training, and open access to updated resources.

## 1. Introduction

The teaching of human anatomy has undergone significant transformations in recent decades, driven by technological advances, innovative pedagogical strategies, and the need to develop durable, safe, and high-quality educational resources. In this context, plastination, developed by Gunther von Hagens in the late 1970s, has become one of the most important techniques for the preservation of anatomical material. This method allows for the replacement of body fluids with curable polymers, stabilizing tissue morphology, eliminating biological risk, and ensuring long-term conservation [1,2,3,4].

Among the various plastination techniques, sheet plastination has stood out due to its usefulness for sectional anatomical study. It provides thin, flat, and translucent specimens that allow for a clear view of the spatial relationships among internal structures. Within this category, polyester-based techniques, notably P35 and P40, have been particularly applied to the central nervous system, offering excellent gray-white matter differentiation, compatibility with radiological imaging modalities, and a format that is ideal for clinical and surgical education [4,5,6].

The P40 technique, developed as an evolution of the P35 method, enables the production of anatomical slices 2–3 mm thick with outstanding transparency and internal detail resolution. Its protocol includes fixation (optional), freezing, dehydration via freeze substitution in acetone at −25 °C, forced impregnation with a resin-catalyst mixture in a vacuum chamber, and finally curing by ultraviolet (UVA) light and controlled heat. Unlike other methods such as the epoxy-based E12 technique, primarily used for thicker slices, P40 is specifically designed to produce thin, semi-transparent sheets in which the tissue is fully integrated into the cured resin layer, providing excellent physical resistance and long-term stability [4].

The technique has evolved from its origins in brain sectioning to the plastination of multiple body regions, thanks to the work of von Hagens and subsequent developments by researchers such as Latorre et al. (2003) [7] and Baptista et al. (2019) [8]. Latorre et al. (2003) [7] proposed P40 as an alternative suitable “for all tissues”, a finding further supported by Baptista et al. (2019) [8] and Ottone et al. (2020) [9] in anatomical education and research contexts. Recent advancements include variations in curing chambers (vertical and horizontal flat chambers), the use of magnets for optimal slice positioning, and strategies to improve resin polymerization and optical clarity even under suboptimal conditions [4,8,9].

Although the technique is well established and has been adopted by institutions across Europe, Asia, and Latin America, its implementation requires specialized equipment, technical expertise, and careful handling, especially during the impregnation and curing stages. Several methodological adaptations have been proposed to improve outcomes, such adjustments to the resin formulation, and modifications to curing conditions to increase efficiency and reduce artifacts. Moreover, its pedagogical advantages have been well-documented: plastinated polyester sections preserve spatial relationships without distortion and provide enhanced correlation with imaging techniques such as CT and MRI, making them highly valued in anatomical and radiological training [10].

In this context of continuous improvement, Gao et al. (2006) [11] introduced a novel variation using Hoffen polyester P45 and a water-bath curing method, applied successfully to the plastination of dolphin slices. Their study demonstrated that the P45 resin, combined with vertical casting chambers and thermal curing, can circumvent the limitations associated with UV light, while maintaining anatomical detail and optical clarity. This technique offers an alternative to traditional P35 and P40 protocols. Moreover, as emphasized by Ottone (2023) [4], the P40 method itself already presented an economic and technical advantage by allowing polymerization under natural conditions, using indirect sunlight at ambient temperatures below 30 °C, thus avoiding the need for costly UV-curing equipment and expanding accessibility for laboratories with limited infrastructure.

In addition to the narrative synthesis, this review incorporates an evaluation of the methodological quality of selected plastination studies using the AQUA tool (Anatomical Quality Assurance), a validated instrument for the appraisal of anatomical research that considers five domains: objectives, study design, methodology, descriptive detail, and reporting of results [12,13]. This is the first manuscript to systematically apply the AQUA tool to plastination research, providing a structured assessment of bias risk and reporting standards in the field.

Given the increasing demand for ethically sustainable, safe, durable, and pedagogically effective anatomical resources, plastination using P40 polyester resin stands out as a valuable tool for modern anatomical education and applied research in clinical anatomy, neuroanatomy, and imaging correlation. Accordingly, the aim of this review is to synthesize and critically analyze the principles, procedures, applications, technical variations, comparative advantages, and current challenges of the P40 plastination technique, situating it within the broader context of polyester-based plastination methods and their impact on contemporary anatomical science.

## 2. Materials and Methods

This study corresponds to a narrative literature review aimed at synthesizing the scientific evidence available on the P40 (Biodur Products GmbH, Heidelberg, Germany) polyester plastination technique and its applications in anatomical preservation. The objective was to explore the origin, technical evolution, methodological variations, educational and research uses, as well as current challenges associated with this technique.

**Search Strategy and Eligibility Criteria**

A structured literature search was conducted between April and August 2025 across three major electronic databases: PubMed, Scopus, and SciELO, with the aim of identifying original research articles focused on polyester-based sheet plastination techniques applied to anatomical preservation, education, or research. The search was performed using the string (“plastination” OR “sheet plastination”) AND/OR (“polyester resin” OR “P40 resin” OR “P35 resin” OR “P45 resin”). In addition, relevant records were also incorporated through manual search.

**Inclusion criteria were as follows:**

- Original research articles published in peer-reviewed journals.
- Studies explicitly addressing polyester-based sheet plastination techniques, including P35 (Biodur Products GmbH, Heidelberg, Germany), P40, or P45 (Dalian Hoffen Biotechnic Co., Ltd., Dalian, China) methods.
- Articles reporting technical innovations, procedural protocols, educational applications, or research use of plastinated specimens.
- Studies conducted on human or animal anatomical specimens.

**Exclusion criteria included:**

- Articles focused exclusively on silicone or epoxy plastination techniques.
- Narrative reviews, editorials, letters to the editor, or conference abstracts without full methodological description.
- Duplicated publications or studies lacking sufficient technical or anatomical detail.
- Studies unrelated to anatomical science or without practical application in education, research, or preservation.

**AQUA Quality Assessment and Reviewer Agreement**

In addition, each included study was critically appraised using structured methodological criteria (AQUA, Anatomical Quality Assurance) [12,13] to evaluate the clarity of objectives, appropriateness of the study design, transparency and reproducibility of technical procedures, anatomical descriptive quality, and relevance of outcomes. This evaluative framework enabled a consistent analysis of the scientific rigor and descriptive depth of the selected literature, contributing to a comprehensive understanding of the robustness and limitations of current evidence on polyester plastination in anatomical science.

The AQUA assessment was independently conducted by two reviewers with expertise in anatomical methodology. Each reviewer scored the included studies across the five AQUA domains (Objectives, Study Design, Methodology, Descriptive Anatomy, and Results Reporting). Any discrepancies were resolved through discussion and consensus; when necessary, a third senior reviewer was consulted to reach a final decision.

## 3. Results

**Figure 1:**
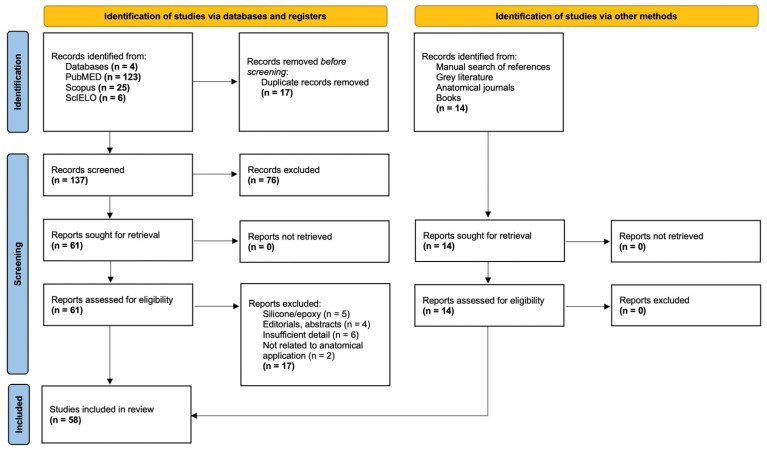
Flow diagram of study selection. From 154 database records and 14 additional records identified manually, 17 duplicates and 93 ineligible studies were excluded. A total of 58 studies fulfilled the inclusion criteria and were included in the review.

The initial search strategy yielded 154 records across PubMed, Scopus, and SciELO. After removing duplicates (*n* = 17) and screening titles and abstracts (*n* = 137 excluded), 61 full-text articles were assessed for eligibility. Of these, 17 were excluded based on the predefined criteria. Additionally, 14 records were identified through manual search, including articles from the Journal of the International Society for Plastination not yet indexed (retrieved from its official website) and two relevant books. Finally, 58 studies met all inclusion criteria and were included in the review (Figure 1). Inter-rater agreement was high, with Cohen’s kappa values ranging from 0.78 to 0.86, confirming the reliability of the scoring process.

The evidence gathered was organized into five main axes: (1) the historical background of polyester plastination, with emphasis on the transition from P35 to P40 techniques; (2) the technical robustness and standardization of the P40 protocol and its methodological adaptations; (3) tissue shrinkage in polyester plastination, addressing its causes, control strategies, and impact on specimen quality; (4) the educational and research utility of P40 plastinated specimens in various anatomical and radiological contexts; and (5) limitations and areas for improvement related to infrastructure, implementation, and protocol dissemination.

The present review confirms that plastination with polyester resin has evolved into a well-established and highly valuable technique for anatomical preservation, particularly in the production of translucent, structurally accurate sectional specimens. Several key themes emerge from the reviewed literature, including the technique’s reproducibility, educational impact, adaptability to various tissues, and ongoing methodological innovations.

## 4. Discussion

### 4.1. Historical Background of Polyester Plastination

**Figure 2:**
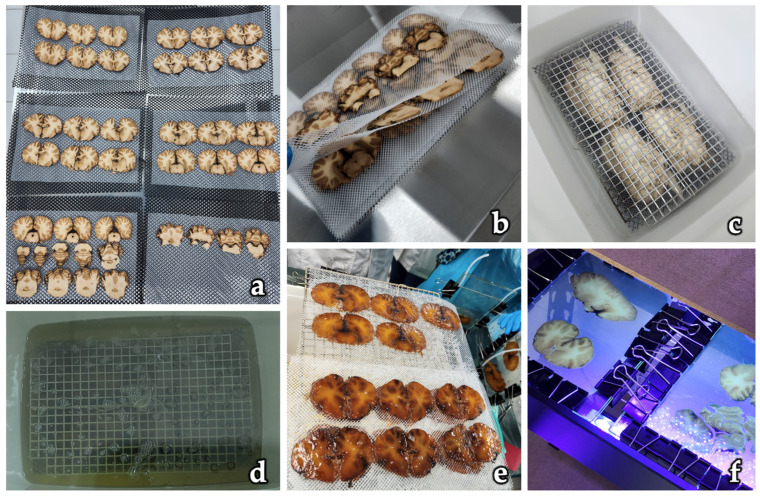
Steps of the polyester sheet plastination technique (P40) applied to brain slices. (**a**) Coronal brain sections positioned on mesh trays for preparation. (**b**) Arrangement of slices between meshes to facilitate handling during the process. (**c**) Dehydration of slices in a cold acetone bath. (**d**) Forced impregnation with polyester resin under vacuum conditions. (**e**) Cured and resin-impregnated sections displaying translucent and durable properties. (**f**) Final embedding of plastinated slices between glass plates for permanent preservation and educational use. Original figure created by the authors based on laboratory work at the Laboratory of Plastination and Anatomical Techniques, Universidad de La Frontera, Temuco, Chile (Director: Prof. Dr. Nicolás E. Ottone).

The development of plastination by Dr. Gunther von Hagens in 1977 marked a transformative milestone in anatomical preservation [1,2,3,4,9]. Designed to overcome the limitations of conventional wet preparations, such as decomposition, strong odor, and biohazard concerns, plastination involves replacing water and lipids in biological tissues with curable polymers, resulting in dry, durable, and non-toxic specimens. Among the various polymer systems developed, polyester resins (notably P35 and later P40) were introduced specifically for the plastination of brain slices, owing to their ability to provide excellent contrast between gray and white matter [10,14]. Some steps of P40 standard polyester sheet plastination process applied to brain slices are illustrated in Figure 2.

The P35 polyester plastination technique was first introduced by Gunther von Hagens in the late 1980s to produce brain sections [2,3]. Despite the excellent quality and contrast of the specimens, this method was technically demanding and difficult to reproduce consistently. In response, von Hagens developed the P40 resin and technique in 1994, with the aim of creating a more user-friendly approach for producing high-quality brain slices [8]. The same P40 resin was later used to generate high-quality plastinates of thin body slices, expanding its application beyond neuroanatomy [7].

In 2006, Gao et al. [11] introduced a third polyester resin, P45, specifically designed for sheet plastination of body parts. With this, the three main polyester sheet plastination techniques, P35, P40, and P45, became established, each named after the corresponding resin. All techniques share the same core plastination steps: specimen preparation, dehydration by freeze substitution, degreasing (optional), forced impregnation, and UV curing [1,15,16].

The rationale behind the development of P40 stemmed from the need for a polymer system that offered the optical clarity of P35 but avoided its limitations, such as the dual heat and UV curing process and the tendency toward bubble formation. As Barnett et al. (2005) [17] noted, P40 was specifically formulated to produce consistently transparent, rigid slices of brain tissue, enabling high-resolution differentiation of gray and white matter. This innovation was driven by growing demands for museum-quality specimens that were easy to handle, durable, and suitable for both education and public display. Furthermore, the authors emphasized that P40’s single-step UV curing and lower viscosity facilitated broader dissemination and standardization of the plastination technique in teaching institutions worldwide.

Early reports demonstrated that polyester plastinates offered superior optical clarity compared to silicone-based specimens [14], making them particularly suitable for neuroanatomy education and radiologic correlation. The P35 technique, although effective, required a dual curing system (UV and heat) and the use of thick safety glass, making it technically complex [15,18]. These limitations prompted the development of the P40 resin, which simplified the curing process and improved resin handling characteristics [8,18]. Unlike epoxy resins such as E12, which provide high transparency but are prone to yellowing and are more labor-intensive, P40 offers a practical balance of clarity, durability, and ease of use. Additionally, P40 can be used without its A4 catalyst, making it more affordable and long-lasting when stored correctly [8].

While P35 remains the gold standard for gray/white matter contrast in brain slices, and P45 is optimized for whole-body cross-sections, P40 is now widely recognized as the most user-friendly and versatile polyester plastination method [5]. Its adoption has greatly facilitated the creation of anatomical teaching collections around the world, contributing to improved diagnostic imaging correlation and anatomical education.

Over the past three decades, these innovations have laid the groundwork for the widespread adoption and continual refinement of polyester plastination protocols in academic and clinical settings.

### 4.2. Technical Robustness and Standardization

The P40 plastination technique demonstrates a high degree of standardization and reproducibility, as initially defined by von Hagens (1987) [3] (Table 1) and subsequently optimized by several researchers. The foundational protocol from which P40 evolved is the P35 polyester plastination technique [6], which produced translucent 4–8 mm slices in flat-chamber systems with exceptional sectional fidelity for brain tissue. Developed through the classic steps of specimen preparation, cold acetone dehydration, impregnation, and curing within glass molds, it served as the gold standard for diagnostic image correlation with CT, MRI, and ultrasound [6]. The familiarity with this method provided a solid technical baseline that guided the optimization of P40. Key improvements included reducing slice thickness to 2–4 mm, which enhanced resolution and handling, improving clarity and gray–white matter contrast, and introducing a simpler curing process under UV light while maintaining reproducible slice quality. In addition, P40 resin exhibits lower viscosity compared to P35, facilitating deeper impregnation and greater transparency.

A further innovation in polyester plastination was introduced by Gao et al. (2006) [11], who developed the P45 technique using Hoffen polyester resin. This method was applied to plastinate large animal specimens, such as a cape dolphin, with highly detailed sectional results. Unlike the P35 and P40 techniques, which rely on UV curing, the P45 technique employs a heated water bath at 40 °C for three days to cure the resin. This water-based curing method ensures uniform temperature distribution, minimizes overheating risks, and offers a cost-effective, safe alternative to UV exposure.

The casting procedure involves a flat chamber made of 5.0 mm tempered glass sealed with 4.0 mm latex or silicone tubing and clamps. Forced impregnation is carried out under vacuum at room temperature, gradually reducing pressure to 0 mmHg. The resin mixture includes 1000 mL Hoffen P45 polyester, 10 g of P45A, 30 mL of P45B (hardener), and 5 g of P45C (plasticizer). The mixture should be freshly prepared and refrigerated to delay thickening. After filling the chamber with the resin, air bubbles are manually removed using 1 mm stainless steel wire. Dehydrated slices are aligned within the chamber, then cured while immersed upright in a circulating 40 °C water bath. This system allows better control of exothermic reactions and eliminates the need for UV lamps or ventilators [19].

Further studies have expanded the spectrum of polyester plastination beyond the traditional P40. Valenzuela et al. (2012) [20] demonstrated that P-4 resin produced rigid and durable thin sections superior to silicone, with improved transparency when used without catalyst and with the additional advantage of resin reusability, thereby reducing costs. More recently, Juvenato et al. (2023) [21] evaluated domestic Brazilian resins as alternatives to P40, showing that P18 produced brain slices of equivalent transparency, stiffness, and white–gray matter differentiation, with shrinkage rates statistically indistinguishable from P40. In contrast, other local polyesters (Cristalan C1–C3) proved unsuitable due to rapid curing and poor handling. Together, these findings indicate that alternative polyesters such as P-4 [20] and P18 [21] can offer cost-effective, reproducible, and sustainable options for plastination while maintaining the morphological fidelity required for teaching and research.

Sui and Henry (2015) [19] further refined the P45 protocol to accommodate both brain and body slices, confirming its suitability for 2–3 mm thick sections. They reported that curing in a circulating water bath not only stabilized temperature uniformly but also accelerated the workflow and simplified quality control. The Hoffen P45 method uses less resin than previous protocols and integrates impregnation and casting in a single system, reducing costs and processing time. The final plastinated slices are semi-transparent, durable, and compatible with radiological correlations (CT, MRI).

Earlier versions of polyester plastination, particularly the P35 technique, required more complex curing systems (UV + heat) and double-glass chambers using safety glass [15,18]. P40 improved upon this by enabling simpler, UV-only curing and the use of single float-glass units, thus reducing costs and technical barriers for implementation [15,16]. Furthermore, the lower viscosity of P40 compared to P35 facilitates deeper penetration and better tissue impregnation, contributing to superior clarity and easier handling.

Moreover, a notable economic advantage of the P40 method lies in its curing flexibility. As indicated in Advances in Plastination Techniques [4], polymerization can be achieved under natural conditions, using indirect sunlight at ambient temperatures below 30 °C, thus eliminating the need for costly UV equipment. This feature makes the technique more sustainable, affordable, and accessible for laboratories with limited resources, while still ensuring high-quality plastinated sections.

A comparative summary of the main polyester sheet plastination protocols (P35, P40 and P45), including their resin type, slice thickness, curing process, advantages, disadvantages, and common applications, is presented in Table 2.

Henry & Latorre (2007) [5] detailed the Biodur™ P40 protocol as a simplified and less equipment-intensive alternative to P35, producing 2–3 mm translucent brain slices with vivid sectional anatomy. They emphasized that P40 requires two-thirds less resin volume, eliminates the need for separate impregnation baths, and allows reuse of the resin bath without loss of quality. These adaptations significantly reduce material costs and simplify processing, making P40 accessible for laboratories with limited resources. In this way, Latorre & Henry (2007) [5] extended the application of the P40 protocol to 2–3 mm semi-transparent body slices, demonstrating that the same core methodology used for brain slices could be successfully scaled to larger anatomical regions, including cat thoracic and abdominal sections. They reported that P40 body plastinates preserve natural tissue color and spatial relationships, making them excellent tools for teaching sectional anatomy and correlating with CT, MRI, and ultrasound. Importantly, the authors emphasized that the P40 technique maintains its advantages, reduced resin consumption, minimal equipment needs, and durability, when applied to body tissue, supporting its versatility beyond neuroanatomical applications.

These technical advantages are particularly significant in the study of complex neuroanatomical structures at the craniovertebral junction. A recent study by Li et al. (2023) [22] described the occipito-atlantal cistern as a distinct and consistent subarachnoid space, located dorsally to the medulla oblongata and enclosed by the posterior atlanto-occipital membrane and dura mater. Its recognition as an anatomically independent compartment has implications for understanding cerebrospinal fluid (CSF) dynamics, as well as for neurosurgical approaches in this region. The ability of polyester plastination to preserve such delicate compartments with spatial accuracy supports its value in advanced neuroanatomical research and education, facilitating the study of fluid-filled spaces and their relationships to surrounding tissues in a preserved, sectioned state.

A significant contribution to the refinement of polyester sheet plastination techniques was provided by Guerrero et al. (2019) [23], who successfully developed a protocol using Biodur^®^ P40 resin for 3 mm human brain slices. Their protocol included a stepwise fixation process with increasing concentrations of formalin up to 20%, followed by dehydration in 100% acetone at −25 °C and a modified active-passive vacuum impregnation cycle at room temperature. This approach enabled cost-efficient resin reuse without catalyst addition, while still producing plastinates with excellent morphological preservation and gray-white matter differentiation. The authors emphasized that insufficient fixation may lead to orange discoloration artifacts when catalyst is used. Moreover, they confirmed that UV-based curing should be performed under controlled temperature conditions (≤30 °C) to prevent glass damage. Finally, the technique was noted to be adaptable beyond neuroanatomy, with careful consideration of polyester-induced tissue shrinkage in other anatomical regions.

However, despite its robustness, the P40 technique remains technically demanding. It requires control over variables such as resin viscosity, slice orientation, vacuum intensity, and UV exposure, which can affect the final optical and structural quality. As reported by Baptista et al. (2019) [8] and Ottone (2023) [4], technical modifications, including vertical curing chambers, repositioning tools, and real-time resin monitoring, can mitigate these challenges and improve workflow efficiency. These refinements, while beneficial, are not yet uniformly adopted, suggesting a need for updated international guidelines or consensus protocols.

Additional refinements to fixation and impregnation protocols have also been proposed. Barnett (1997) [24] emphasized the importance of prolonged and uniform fixation, particularly at low temperatures (+5 °C), using repeated formalin baths over 9 to 11 weeks. This step was found to be essential in preventing tissue discoloration (e.g., orange cortical spots), likely due to residual peroxidase activity interacting with the P40 catalyst. Furthermore, to stabilize tissue integrity and reduce white spot artifacts during dehydration, careful bubble release and frequent repositioning of slices during cold acetone baths were recommended. Vacuum impregnation at room temperature, combined with cooling of the polymer bath using ice packs, was shown to ensure consistent resin penetration while avoiding premature polymer warming and polymerization.

A noteworthy consideration in polyester plastination protocols, specifically P35, is the impact of extended immersion duration on specimen quality. A technical report by Üzel & Weiglein (2013) [25] described brain slices unintentionally left for two years in the second immersion bath (P35/A9 mixture) at +5 °C. Despite this prolonged period, the final plastinates maintained satisfactory optical quality. However, significant mechanical issues were observed, including increased fragility, partial curing, adhesion of filter papers, and difficulty in manipulation due to elevated resin viscosity and localized pre-polymerization. These findings indicate that while immersion duration can be extended for logistical flexibility, plastinators must closely monitor the physical state of the resin and avoid excessive storage times. Prolonged contact with metal grids or filter materials should also be minimized to prevent deformation or adherence artifacts. The report suggests that immersion can last weeks or even months without compromising visual outcomes, but mechanical complications should be anticipated and managed accordingly.

A marked variation in the dehydration step was proposed by Sora and Brugger (2000) [26], who explored the use of methanol as a substitute for acetone to reduce handling risks associated with acetone’s volatility and low flash point. Using technical-grade methanol cooled to −20 °C, they demonstrated successful plastination of human brain slices without compromising structural integrity or visual clarity when compared to acetone-based dehydration. Although the authors noted no macroscopic differences in the final plastinates, they emphasized that methanol requires precise vacuum control during impregnation, as its extraction occurs within a narrow pressure range (between 92 and 61.5 mmHg). Additionally, they highlighted that only experienced plastinators should attempt this method due to the need for careful monitoring of residual solvent removal. This approach offers a safer, cost-effective alternative for dehydration in laboratories with strict chemical safety regulations.

Additional contributions by Henry & Latorre (2007) [5] emphasize the importance of proper slice fixation before resin impregnation, particularly for thin or delicate tissues. Their use of pinning slices to polyethylene foam boards during dehydration and impregnation ensured minimal distortion and facilitated handling. Moreover, their adaptations of the curing chamber, such as the use of flat acrylic plates with adjustable spring clamps, enabled effective curing without bubble entrapment or resin leakage, especially in large-format slices. These procedural innovations are especially relevant for plastination centers with limited resources or without access to custom-built curing molds.

Precise curing duration and temperature control are critical for P40 plastinates. According to Reed et al. (2008) [27], when using UVA light (four 40 W bulbs positioned 35 cm from the chamber), a minimum curing time of one hour is required; however, actual needs vary depending on lamp wattage, distance, and specimen size. They emphasize active cooling, via fans or compressed air, to maintain glass temperatures below 30 °C, preventing exothermic damage to the tissue. Natural daylight (indirect sunlight) has also been validated as an effective and cost-free curing source, though it necessitates periodic chamber repositioning to ensure uniform exposure and avoid shrinkage or cracking due to rapid polymerization.

### 4.3. Tissue Shrinkage in Polyester Plastination

The addition of quantitative assessments of tissue shrinkage [8] provides important insights into one of the few disadvantages of the technique. Reported shrinkage ranges between 7 and 10% after impregnation, which is considered acceptable when compared to other plastination methods, especially when conducted at low temperatures. In support of this, Sora et al. (1999) [28] experimentally demonstrated that conducting both immersion and impregnation at −25 °C significantly reduced linear and surface shrinkage (1.92% and 4.41%, respectively), in comparison to higher temperatures (2.60% and 6.96% at +15 °C). These results highlight the importance of temperature control to minimize morphological deformation, particularly in delicate tissue slices used for morphometric studies.

Related to P45, reported shrinkage ranges from 2 to 8%, with a refractive index of 1.49, indicating excellent preservation of tissue morphology [29]. A subsequent quantitative study by Okoye, Dou, and Sui (2019) [30] evaluated the extent of tissue shrinkage in various organs following P45 plastination. Using digital imaging before and after plastination, they found that shrinkage varied according to tissue type: cerebral cortex exhibited the lowest mean shrinkage at 6.39% (±3.9), followed by kidney (8.26%), muscle (6.73%), and liver (10.2%). In contrast, spleen and lung tissues showed greater shrinkage, with lungs exhibiting the highest at 19.86% (±1.68). These findings suggest that both tissue composition and physical properties, such as elasticity and cellular density, significantly influence shrinkage rates.

In another study, Ottone et al. (2020) [9] analyzed tissue shrinkage specifically in 3 mm human brain slices plastinated using the Biodur^®^ P40 polyester resin. The specimens were fixed with formalin, dehydrated in 100% acetone at −25 °C, and subjected to forced vacuum impregnation at 20 °C followed by UV curing. Shrinkage was measured in both lateral-lateral and superior-inferior directions. After dehydration, shrinkage ranged from 2.14% to 6.22%, while after forced impregnation it increased to 7.02–10.62%. These findings confirm that most shrinkage occurs during the impregnation step.

Ottone et al. (2020) [9] also highlighted that shrinkage levels are influenced by tissue orientation and direction of measurement, being slightly higher in lateral-lateral than superior-inferior measurements. The statistical analysis employed Shapiro–Wilk, Wilcoxon, and Student’s *t*-tests, confirming significant differences (*p* < 0.05) across stages of the plastination process. These results are consistent with previous observations and reinforce the recommendation to use low-temperature dehydration protocols and precise slicing techniques to reduce morphological distortion.

The authors emphasized that although P45 plastination is effective for producing semi-transparent and morphologically accurate specimens, morphometric and 3D reconstructions must account for shrinkage and the mechanical loss of material during sawing [8]. Their results align with earlier observations on shrinkage behavior in P40 and E12 techniques, reinforcing the importance of standardized conditions (e.g., dehydration at −25 °C, control of degreasing time, and consistent impregnation protocols) to minimize distortion and preserve structural fidelity.

### 4.4. Educational and Research Utility

A consistent finding across multiple studies is the exceptional utility of P40 plastinated slices in anatomical and clinical education (Table 3). These specimens offer unmatched clarity in demonstrating the spatial relationships between structures, particularly in neuroanatomy, head and neck anatomy, and thoracic sections. The translucency of the resin and sharp differentiation of gray and white matter facilitate learning and allow for direct correlation with radiological images [10].

Similarly, Wadood et al. (2001) [14] reported that polyester-copolymer impregnated brain slices displayed a distinctly superior contrast between gray and white matter compared to silicone plastinates. This contrast enhances both anatomical interpretation and correlation with neuroimaging, making these plastinates especially useful for teaching neuroanatomy and radiological anatomy in academic settings.

Weiglein (1996, 1997) [16,31] showed that P35/P40 plastinated slices were not only preferred by students over formalin-fixed specimens but also significantly improved learning outcomes in neuroanatomy. They allowed integration of anatomical, radiological, and topographic knowledge without exposure to toxic substances or tissue deterioration. Furthermore, Weber & Henry (1992) [15] detailed the effectiveness of 4 mm brain slices impregnated with P35/P40, cured under controlled UV and heat conditions, to produce long-lasting, structurally accurate models. Unlike traditional wet preparations, P40 plastinates are dry, odorless, durable, and biosecure, attributes highly valued in modern teaching environments and museum displays.

Additionally, Latorre & Henry (2007) [32] highlighted that body plastinates produced with P40 retain vivid sectional anatomy and natural coloration, which enhances their educational impact by facilitating direct correlation with imaging studies such as CT, MRI, and ultrasound. These extended-format plastinates, thus, represent powerful tools for teaching complex regional anatomy in clinical, radiological, and veterinary curricula.

From a research perspective, the technique also contributes to morphometric studies, particularly in brain anatomy, where slice thickness and orientation can be precisely controlled. Studies such as Ottone et al. (2020) [9] have quantitatively assessed tissue behavior throughout the plastination process, generating valuable data for both anatomical validation and technique optimization.

Recent applications have extended to veterinary anatomy as well. Latorre et al. (2003) [7] demonstrated the combined use of S10 and P40 plastinated slices for the interpretation of MR images of the equine tarsus. P40 slices enabled enhanced visualization of muscular and ligamentous structures, especially in transverse sections, which were difficult to discern in MR alone. This study illustrates the synergistic value of P40 plastinates in diagnostic anatomy and comparative imaging.

The potential of P40 plastination for precise morphometric analysis was notably demonstrated by Genser-Strobl and Sora (2005) [33], who used P40 plastinated frontal slices of the human hip joint to obtain accurate measurements relevant to orthopedic surgery and implant planning. Their findings showed strong concordance with conventional 3D measurements, particularly in bone structures such as the femoral head and neck. Importantly, they validated the utility of P40 slices for capturing geometrical data with minimal shrinkage, reinforcing plastination’s value in studying complex joint morphology and enhancing preoperative assessments for procedures like total hip arthroplasty.

Recent studies using the P45 sheet plastination technique have significantly advanced our understanding of musculoskeletal, neuroanatomical, and connective tissue structures across species. In the context of biomechanical anatomy, Jiang et al. (2021) [34] demonstrated that the fibula plays a critical supporting role in the lateral tibial plateau through an arch-like trabecular system, offering a biomechanical explanation for nonuniform tibial settlement in knee osteoarthritis. Complementing this, Jiang et al. (2022) [35] revealed that trabecular bone at the attachment sites of the cruciate ligaments is organized according to mechanical loading patterns, radial, parallel, and longitudinal, providing valuable insights into ligament reconstruction strategies. Sun et al. (2021) [36] further mapped the cancellous bone architecture of the proximal tibia, identifying three principal load-bearing regions that align with the mechanical stress pathways of the knee joint. In addition, Cheng et al. (2025) [37] used 3 mm P45 plastinated knee sections to investigate the anterior attachments of the medial patellofemoral ligament (MPFL), showing that it does not attach directly to the patella but instead integrates with the vastus medialis obliquus, vastus intermedius, and parapatellar tendons. These findings highlight the capacity of plastination to reveal novel anatomical details with both educational and clinical relevance, particularly for optimizing MPFL reconstruction procedures. Extending this approach to the hip, Shah et al. (2025) [38] combined P45 plastination with three-dimensional reconstruction and finite element analysis to characterise the trabecular systems of the proximal femur, demonstrating that vertical trabeculae primarily absorb compressive loads while horizontal trabeculae resist tensile forces, with Ward’s triangle emerging as a structurally vulnerable region. Their results underscore the dual value of plastination in clarifying trabecular architecture for educational purposes and informing implant design in orthopaedic practice.

In the realm of surgical and clinical anatomy, Qin et al. (2021) [39] offered a comprehensive delineation of the medial and lateral canthal ligaments, revealing a hammock-shaped orbital suspensory ligament that segments postseptal fat and plays a key role in supporting the lower eyelid, findings of high relevance for oculoplastic interventions. Similarly, Ma et al. (2020) [40] examined the retro-orbicularis oculi fat (ROOF) and confirmed its layered distribution and anatomical continuity, supporting its clinical relevance in periorbital surgery. Chun et al. (2015) [41] also demonstrated the utility of P45 plastination for high-resolution visualization of orbital soft tissues, including the preseptal and preaponeurotic fat compartments in the eyelid, reinforcing the method’s value in both neuroanatomical and surgical morphology. Zhang et al. (2017) [42] extended the application of this technique to the perineal region by clarifying the spatial boundaries and anatomical contents of the ischioanal fossa, improving the interpretation of perineal imaging and surgical access. Building on this, Zhang et al. (2025) [43] systematically analysed P45 plastinated sections of the proximal femur in multiple planes, describing a “mushroom-like” configuration of the primary compressive strut and redefining Ward’s triangle as a quadrangular pyramidal space separating two independent trabecular subsystems. This refined spatial model not only enhances anatomical education but also provides critical guidance for improving internal fixation methods, orthopaedic implants, and future biomechanical simulations.

Recent anatomical studies have highlighted the structural basis of facial grooves and their relevance in aesthetic procedures. Hwang et al. (2021) [44] showed that the nasolabial fold aligns with the medial edge of the superficial fascia, supporting targeted subcutaneous or sub-SMAS dissection. Du et al. (2021) [45] identified fibroelastic bundles beneath the orbicularis oculi muscle as key in midcheek groove formation, with deep filling yielding significant aesthetic improvements. These findings underscore the importance of fascia and fibromuscular architecture in facial contouring strategies.

P45 plastination has also proven to be a powerful tool in elucidating the morphology and significance of the myodural bridge (MDB) and related craniovertebral structures. Zheng et al. (2017) [46] confirmed the universal presence of the MDB across multiple mammalian orders, suggesting it as a homologous and evolutionarily conserved structure essential to suboccipital dural dynamics. In marine mammals such as Neophocaena phocaenoides and the sperm whale, the MDB was likewise consistently identified by Zhang et al. (2021) [47], indicating functional conservation in aquatic environments, possibly contributing to cerebrospinal fluid (CSF) regulation during diving. Yuan et al. (2016) [48] classified four distinct MDB attachment patterns originating from the rectus capitis posterior minor muscle, including connections to the atlas and adjacent dural spaces, providing insight into mechanisms underlying cervicogenic headaches and CSF dynamics. Chi et al. (2022) [49] proposed redefining the MDB as a broader “myodural bridge complex,” comprising multiple muscular and ligamentous connections to the dura mater. This expanded definition highlights the MDB’s potential physiological roles, such as CSF propulsion and stabilization of the cervical dural sac. Chi et al. (2022) [49] also documented how congenital atlanto-occipital fusion alters MDB morphology, identifying compensatory adaptations via the nuchal ligament. Zhang et al. (2016) [50], in turn, described the connection between the occipital muscles and spinal dura in the Siamese crocodile, further demonstrating evolutionary conservation of the MDB across species.

Additional investigations have leveraged P45 plastination to explore deep dural insertions and other functionally significant structures. Zheng et al. (2014) [51] proposed a functional link between the “To Be Named Ligament” (TBNL), the vertebrodural ligament (VDL), and CSF dynamics, emphasizing the role of plastination in visualizing complex dural attachments. Jiang et al. (2023) [52] applied the technique to define the previously ambiguous corpora-glans ligament, a fibrous structure anchoring the corpus cavernosum to the glans penis, essential for penile integrity and reconstruction. Lastly, Zhang et al. (2021) [47] identified a novel muscle, the occipital-dural muscle, in finless porpoises, forming part of the MDB and illustrating how plastination can uncover new anatomical-functional relationships in non-human species. Zhang et al. (2025) [53] used plastinated sections and dissection to define the sphenomandibularis (SM), confirming its distinction from the temporalis muscle and its close relations to major neurovascular structures, with both educational and clinical implications for craniofacial anatomy.

Collectively, these studies underscore the exceptional versatility of P45 plastination in resolving complex anatomical relationships, from trabecular microarchitecture and surgical anatomy to evolutionary neurostructures and soft tissue morphology. The technique continues to bridge the gap between gross dissection and fine-resolution imaging, offering insights that support clinical interventions, evolutionary biology, and functional neuroanatomy alike.

**Table 3:** Summary of anatomical regions explored through polyester sheet plastination techniques, detailing the objectives of each investigation, principal morphological or functional findings, and relevant bibliographic sources. The compilation reflects the methodological versatility of polyester plastination in supporting high-resolution anatomical analysis across diverse systems and species.

Anatomical Region	Purpose of Study	Key Findings/Advantages	Reference(s)
**Human brain (curriculum specimens)**	Use of plastinated brain slices in anatomical education	Implementation of 4 mm P35 brain sections for neuroanatomy teaching to medical and allied health students; offered excellent gray–white matter contrast, cleanliness, odorless handling, durability, and direct correspondence with CT imaging courses	Weiglein (1993) [18]
**Brain (coronal and Horizontal slices)**	Neuroanatomical visualization	Produced thin (4–8 mm) odorless, high-contrast slices between gray and white matter; suitable for teaching without handling constraints	Barnett (1997) [24]
**Human Brain (sagittal slices)**	To determine the effect of different immersion and impregnation temperatures on shrinkage rates in brain plastination	Sagittal 4 mm slices processed at −25 °C (immersion and impregnation) showed lower shrinkage (4.41%) compared to slices processed at +5 °C/+15 °C (6.96%); supports low-temperature processing to enhance morphometric fidelity	Sora et al. (1999) [26,28]
**Human Brain (methanol)**	Evaluation of methanol as alternative dehydration solvent	Successfully plastinated 4 mm coronal brain slices using methanol (−20 to −25 °C) instead of acetone; no processing issues noted; methanol dehydration did not impair final P40 plastinate quality	Sora & Brugger (2000) [26]
**Brain (Coronal slices)**	Neuroanatomical visualization and educational use	Achieved clear, detailed coronal sections of the brain with preserved structural relationships using P35; suitable for teaching without odor or toxicity	Barnett et al. (2005) [17]
**Brain (Coronal slices)**	Neuroanatomical visualization for teaching	Preserved structural relationships; high-quality teaching material with minimal shrinkage	Guerrero et al. (2019) [23]
**Human Brain (3 mm slices)**	To statistically analyse tissue shrinkage during sheet plastination with polyester resin (Biodur^®^ P40)	Reported shrinkage between 2.14 and 6.22% after dehydration and 7.02–10.62% after forced impregnation; confirmed that most shrinkage occurs during impregnation; highlighted need for low-temperature dehydration to reduce distortion	Ottone et al. (2020) [9]
**Knee Joint**	To explore fibular support to the tibial plateau (Proximal tibiofibular region)	Found dense trabecular connections forming an arch-like structure that may influence knee load distribution and KOA	Jiang et al. (2021) [34]
	To analyze bony features at cruciate ligament attachment sites	Identified dense ridges and trabecular structures supporting ligament anchorage and guiding reconstruction	Jiang et al. (2022) [35]
	To clarify the anterior insertions of the medial patellofemoral ligament (MPFL)	Demonstrated that the MPFL does not attach directly to the patella but integrates with the vastus medialis obliquus, vastus intermedius, and parapatellar tendons; findings refine surgical reconstruction strategies	Cheng et al. (2025) [37]
**Proximal Tibia (Knee joint)**	Trabecular architecture mapping	Identified trabecular patterns: thick longitudinal trabeculae in condyles, network in intercondylar region, arcuate trusses in metaphysis; reinforced bone near ligament attachments	Sun et al. (2021) [36]
**Proximal femur**	To investigate trabecular morphology and biomechanics via plastination and finite element analysis	Identified medial and lateral trabecular systems; vertical struts absorb compression, horizontal struts resist tension; Ward’s triangle defined as structurally weak region with clinical relevance for implant design	Shah et al. (2025) [38]
	To qualitatively analyse the three-dimensional arrangement of trabeculae in coronal, sagittal, and horizontal planes	Described a “mushroom-like” primary compressive strut and two independent trabecular subsystems (head–neck and neck–shaft), separated by a redefined quadrangular Ward’s triangle; insights inform fixation, implant design, and surgical robotics	Zhang et al. (2025) [43]
**Craniovertebral junction/Miodural bridge**	Definition of TBNL and VDL	Proposed link to CSF circulation; clarified dural attachments	Zheng et al. (2014) [51]
	To assess MDB presence across mammalian species	Found MDB in all 15 species, suggesting it is a conserved and essential structure	Zheng et al. (2017) [46]
	To classify the attachment patterns of the myodural bridge from the RCPmi muscle	Identified four insertion patterns of the myodural bridge; supports its role in CSF dynamics and cervicogenic headache pathophysiology	Yuan et al. (2016) [48]
	To define the MDBC as a functional anatomical unit	Identified MDBC components (RCPmi, RCPma, OCI); proposed roles in CSF flow, dural tension, and venous return	Zheng et al. (2020) [54]
	To examine effects of congenital C0–C1 fusion on the MDB	Found that MDB persists via nuchal ligament fibers despite absence of key muscles, indicating compensatory support	Chi et al. (2022) [49]
	To analyze the fiber composition of the cervical spinal dura mater	The SDM is a three-layered structure formed by fibers from the cerebral dura, occipital periosteum, and myodural bridge; findings support dural preservation in CVJ surgery	Zhuang et al. (2022) [55]
	To study LCT structure via P45 and confocal microscopy	Revealed layered collagen architecture and tissue connections; useful for lateral canthoplasty	Li et al. (2023) [22]
	To analyze the detailed morphology of the suboccipital cavernous sinus (SCS) and its anatomical relationship with the myodural bridge complex (MDBC)	Identified fibrous MDBC connections to the SCS; suggests role in venous drainage and intracranial pressure regulation.	Zhang et al. (2023) [56]
**Orbit/Eyelid**	To study fat tissue distribution (upper eyelid, Asian)	Clarified preseptal and preaponeurotic fat layers; high-resolution sections for surgical relevance	Chun et al. (2015) [41]
	To analyze ROOF continuity using histology and P45 (periorbital region)	Found ROOF is a continuous fat layer from brow to cheek; relevant for surgery and filler safety	Ma et al. (2020) [40]
	To examine canthal ligaments (periorbital region)	Revealed structure and insertions of canthal ligaments; useful for eyelid surgical planning	Qin et al. (2021) [39]
**Nasolabial region**	To study the relationship between nasolabial fold and superficial fascia	Found nasolabial fold aligns with medial edge of SFS; supports surgical approaches for facial rejuvenation	Hwang et al. (2021) [44]
**Midcheek region**	To analyze anatomical basis of midcheek groove and evaluate treatment	Identified fibroelastic bundles beneath OOM linked to groove; deep filling improved clinical outcomes	Du et al. (2021) [45]
**Infratemporal and pterygopalatine fossae (masticatory muscles)**	To characterise the debated sphenomandibularis (SM) using plastination and dissection	Confirmed distinct morphology from temporalis; close relation to maxillary nerve, buccal nerve, and maxillary artery; supports recognition of SM as independent muscle with educational and clinical relevance (neuralgia, nerve block, mandibular biomechanics)	Zhang et al. (2025) [53]
**Perineum**	Spatial organization of ischioanal fossa	Improved interpretation for imaging and surgery	Zhang et al. (2017) [42]
**Penis**	Definition of corpora-glans ligament	Clarified anatomical anchoring relevant to reconstruction	Jiang et al. (2023) [52]
**Hip Joint**	Morphometric validation for orthopedic planning	Agreement with 3D metrics; minimal shrinkage	Genser-Strobl & Sora (2005) [33]
**Comparative anatomy (mammals, reptiles)**	MDB structure across species	Demonstrated universal presence and functional conservation	Zhang et al. (2016) [50]
**Sperm whale**	Aquatic adaptations of MDB	Suggested CSF regulation during diving	Liu et al. (2018) [57]
**Neophocaena**	Characterize the myodural bridge as independent muscle	Identification of an independent muscle (“occipital-dural muscle”) potentially involved in cerebrospinal fluid circulation	Zhang et al. (2021) [47]
**Equine Tarsus (Horse ankle)**	Aid in interpreting MR images via plastinated slices	P-40 semitransparent and S10 thick slices (2–10 mm) provided detailed anatomical correlation with sagittal and transverse MR images, improving visualization of joints, tendons, synovial pouches and muscle–tendon relationships	Latorre et al. (2003) [7]
**Suboccipital region (marine mammal)**	To verify the existence and structure of the myodural bridge (MDB) in Neophocaena phocaenoides	The MDB exists in this species; RCDmi inserts into the cervical spinal dura via a tendinous bridge through the posterior atlanto-occipital interspace. No dorsal atlanto-occipital membrane was observed. P45 plastination and histology confirmed the structure, composed of type I collagen	Liu et al. (2017) [58]
**Multiple tissues (brain, viscera, bone, muscle)**	To present polyester sheet plastination as a versatile method applicable to different tissue types.	Technique produced durable, odorless, and transparent plastinated sheets across multiple organs and systems; emphasized its adaptability and suitability for both teaching and research, expanding applications beyond neuroanatomy.	Latorre et al. (2004) [59]

### 4.5. AQUA-Based Analysis of Anatomical Studies

**Figure 3:**
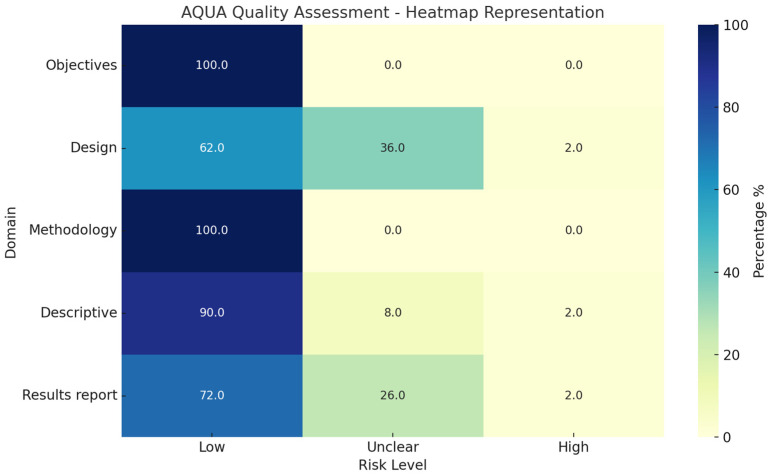
Heatmap representation of the AQUA (Anatomical Quality Assurance) assessment [12,13], applied to studies on polyester sheet plastination. Percentages of studies rated as Low, Unclear, or High risk are shown for each of the five AQUA domains: Objectives, Study Design, Methodology, Descriptive Anatomy, and Results Reporting. The heatmap highlights the consistently low risk in Objectives and Descriptive Anatomy, while Study Design and Results Reporting display higher proportions of unclear or high risk.

A comprehensive evaluation of the methodological quality of anatomical research on polyester sheet plastination was carried out using the AQUA (Anatomical Quality Assurance) tool [12,13], and the results are summarized in a heatmap representation (Figure 3). This analysis, applied to peer-reviewed articles, allowed classification according to five domains, Objectives, Study Design, Methodology, Descriptive Anatomy, and Results Reporting, and facilitated the identification of evolving trends in anatomical research practices and scientific writing over time. In addition to the aggregate percentages and the heatmap presented, the individual scoring of each included study across the five AQUA domains (Objectives, Study Design, Methodology, Descriptive Anatomy, and Results Reporting) has been compiled in Supplementary Table S1 (see Supplementary Materials). This table presents the methodological quality of each study in detail and complements the overall trends, thereby ensuring transparency in the assessment process.

In general, the clarity of objectives (Domain 1) was consistently adequate across the time span, with most studies clearly articulating their goals, whether technical or anatomical. Earlier articles from the 1980s and 1990s tended to present objectives more broadly and with less contextual framing, while more recent publications, especially from the past decade, have increasingly incorporated structured rationales and contextualized their aims with literature-based justifications. This reflects a gradual alignment with contemporary research expectations. Notably, the AQUA analysis confirmed this trend, with 100% of studies classified as Low risk in this domain.

For study design (Domain 2), the analysis revealed more notable differences over time. Early studies often lacked detailed descriptions of sample selection, control variables, or replication strategies, which resulted in a higher proportion of studies assessed as having unclear or potentially higher risk of bias in this domain. Nevertheless, a progressive shift was noted in articles published since the mid-2000s, with more frequent inclusion of specimen counts, inclusion criteria, and ethical considerations. Although formal designs with statistical planning, randomization, or comparison groups remain limited, especially in technical reports, there is evidence of an increasing concern for methodological coherence. Consistent with this, the heatmap results show that 62.1% of articles were Low risk, 34.5% Unclear, and 3.4% High.

In the domain of methodology (Domain 3), improvements were particularly marked. Initial publications focused on the foundational establishment of plastination techniques and often lacked standardized terminology or consistent environmental controls. Over time, however, methodological descriptions have become more detailed and reproducible, with increased inclusion of controlled variables, stepwise procedures, and quality control parameters. Recent studies on polyester plastination, especially those published after 2010, frequently integrate complementary methodologies such as histology, morphometry, and 3D imaging, reflecting an overall advancement in experimental sophistication and rigor. This aligns with the AQUA results, where 93.1% of studies were rated Low risk.

Descriptive anatomy (Domain 4) remained a core strength throughout the timeline. From early works to more recent publications, anatomical studies have generally shown accurate structural depiction and effective use of visual documentation. That said, recent articles have benefited from enhanced technologies, including high-resolution imaging, digital illustration, and standardized anatomical terminology. These additions have contributed to greater clarity, reproducibility, and educational value. The AQUA analysis reflects this strength, with 89.7% Low risk and only 10.3% Unclear.

The most substantial progression was observed in the reporting of results (Domain 5). Older articles tended to provide narrative descriptions without quantitative support, figures, or structured tables. In contrast, contemporary publications demonstrate an increasing tendency to include tabulated findings, measurements, frequency reporting, and, in some cases, basic statistical analysis. Nonetheless, there remains room for improvement in the systematic reporting of variability, effect sizes, and confidence intervals. The domain, although evolving positively, continues to show variability in reporting standards. This is consistent with the AQUA results, which highlight 51.7% Low risk, 41.4% Unclear, and 6.9% High in this domain.

When examining article types, technical reports, particularly those detailing plastination procedures, showed considerable improvements in protocol clarity and process control, albeit with less emphasis on formal study design and statistical validation. Descriptive anatomical studies, in turn, consistently demonstrated strong observational quality, with increasingly robust methodological integration and reporting clarity in the most recent publications.

This longitudinal analysis highlights a clear trajectory of progressive enhancement in methodological quality, scientific rigor, and alignment with evolving academic standards. While early studies were exploratory in nature, aimed at establishing procedures and describing novel anatomical insights, more recent publications reflect a broader commitment to reproducibility, interdisciplinary integration, and standardized reporting practices. Importantly, the AQUA assessment identified relatively few instances of high risk of bias, with most domains falling within Low or Unclear categories, suggesting an overall moderate-to-high methodological standard across the reviewed literature.

These results underscore the relevance of structured evaluation tools like AQUA not only for retrospective assessment but also as useful frameworks for guiding research planning, peer review, and editorial decision-making. As the discipline of anatomical science continues to evolve, the increasing adoption of such tools may support further improvements in methodological transparency while preserving the descriptive richness that defines the field.

### 4.6. Limitations and Areas for Improvement

Despite its numerous advantages, P40 plastination is not without limitations. The technique is resource-intensive, requiring specialized equipment, safe handling of volatile chemicals such as acetone, and trained personnel. The toxicity of resins and solvents demands strict occupational safety measures, including adequate ventilation, fume extraction systems, and the use of personal protective equipment, since prolonged exposure can cause irritation and systemic health effects [4,16,24]. Moreover, the polymerization process can be unpredictable if resin temperature, UV intensity, or curing duration are not carefully controlled, with studies reporting instances of incomplete curing, resin opacity, or bubble formation when conditions deviate from the optimal range [4,9]. One specific challenge reported by Barnett (1997) [24] is the occurrence of orange cortical discoloration in brain slices derived from conventionally fixed specimens, underscoring that P40 plastination is more sensitive to fixation quality than S10 or P35 and requires tailored fixation protocols to achieve optimal results.

A further limitation is related to access and dissemination. Implementation in low-resource settings remains difficult due to the high costs of specialized resins, vacuum pumps, curing equipment, and controlled freezers, restricting its use mainly to well-funded institutions [7]. Nevertheless, as noted by Ottone (2023) [4], the technique can be successfully adapted and optimized in institutions with greater resources, where investment in infrastructure and training ensures reproducibility and high-quality results. In parallel, limited dissemination of updated protocols hinders broader implementation, as many innovations remain confined to local experiences or workshop settings. Establishing open-access repositories and training programs will, therefore, be essential to promote equitable access.

Environmental sustainability also represents a challenge. The disposal of large volumes of acetone and non-biodegradable polymers contributes to chemical waste, highlighting the need for protocols that minimize solvent use and for research into eco-friendly or recyclable resins [20,21]. Additionally, as reported by Latorre et al. (2003) [7], combining P40 with other plastination techniques such as S10 may be necessary to fully represent all anatomical features, since P40 alone is not always sufficient to provide deep contrast or three-dimensional relief.

Finally, the AQUA-based quality assessment of the literature revealed generally favorable outcomes across the five domains evaluated. Most studies clearly stated their objectives and described coherent methodological approaches, with a predominance of low or unclear risk of bias and very few instances categorized as high risk. Technical reports tended to focus more on procedural description, while anatomical investigations showed increasing clarity in study design, descriptive rigor, and alignment between methodology and outcomes. These findings suggest a positive trajectory in the development of plastination research quality over time and support the use of structured evaluation frameworks, such as AQUA, to further enhance methodological transparency, reproducibility, and scientific robustness.

## 5. Conclusions

Plastination with polyester resin has proven to be a robust, reproducible, and pedagogically impactful technique for the preservation of anatomical sections. Its ability to produce translucent, structurally accurate specimens has made it especially valuable in neuroanatomy, sectional anatomy, and the integration of radiological and topographical knowledge. Over the past three decades, the technique has evolved from its early implementations to more refined, user-adaptable protocols, incorporating technical innovations that enhance both the quality of the final product and the efficiency of the process.

Despite certain limitations, such as the need for specialized infrastructure, operator expertise, and meticulous control of variables, the reviewed literature demonstrates that these challenges can be mitigated through methodological adaptations, inter-institutional collaboration, and standardized training. Furthermore, the versatility of P40 plastinates has been highlighted across a wide range of applications, from medical and veterinary education to morphometric and imaging-based research.

The evidence presented supports the continued use and expansion of P40 plastination in academic institutions worldwide. Future efforts should prioritize the dissemination of updated protocols, development of cost-effective equipment, integration with digital platforms, and the promotion of multilingual resources to democratize access to this valuable anatomical preservation technique.

Additionally, the AQUA-based quality assessment conducted in this review revealed a positive trend toward greater methodological rigor, improved reporting standards, and clearer research objectives over time. This underscores the progressive maturation of anatomical literature on polyester plastination and highlights the value of structured evaluation frameworks in guiding future scientific production. As anatomical science continues to embrace technological innovation, P40 plastination stands as a bridge between traditional morphology and modern educational and research paradigms.

## Figures and Tables

**Table 1:** Step-by-step table for P40 sheet plastination technique. Detailed description of the classic polyester sheet plastination technique (Biodur® P40) for anatomical slices. The main protocol steps are listed along with the specific reagents and equipment required for each phase of the process, from fixation and dehydration to impregnation, curing, and final storage.

Step	Description	Reagents	Equipment
**1. Fixation** **(optional)**	Tissue is fixed in 4% formaldehyde or buffered formalin solution to preserve morphology.	4% buffered formaldehyde	Fixation tanks, PPE (gloves, masks, lab coat)
**2. Slicing**	Specimens are frozen and sectioned into slices (2–4 mm) using a band saw or slicing device.	None	Band saw with liquid nitrogen or freezing unit, cryoprotection chamber
**3. Freeze Substitution Dehydration**	Slices are placed in cold 100% acetone at −25 °C to dehydrate tissues while maintaining structure (15 to 20 days)	100% acetone	Deep freezer (−25 °C), acetonometer, dehydration containers
**4. Pre-Impregnation Setup**	Slices are positioned between polyethylene or foam boards to maintain orientation and prevent folding (24 h)	None	Polyethylene foam boards, pins or clamps
**5. Vacuum Impregnation with P40**	Dehydrated slices are submerged in P40 resin under vacuum to replace acetone with polymer (24 h)	P40 polyester resin	Vacuum chamber, vacuum pump, pressure gauge, resin container
**6. Curing (Polymerization)**	Impregnated slices are placed between glass or acrylic plates and cured with UV light.	UV-sensitive P40 resin (no hardener required)	UV light source (e.g., 315–400 nm), glass or acrylic sandwich chambers, clamps or spring systems
**7. Trimming and Cleaning**	Cured slices are removed from the chamber, excess resin trimmed, and surfaces cleaned.	Isopropanol or ethanol for cleaning	Scalpel or blade, soft cloths, trimming tools
**8. Labeling and Storage**	Finished plastinates are labeled and stored in protective cases at room temperature.	None	Archival containers, labeling system

**Table 2:** Comparative features of the three main polyester sheet plastination techniques (P35, P40 and P45). The table summarizes the type of resin used, typical slice thickness, curing process, major advantages and disadvantages, and common applications of each protocol. P35 provides superior gray-white matter contrast but is technically demanding; P40 is the most versatile and widely adopted method due to its transparency and simplified curing; and P45 offers a cost-effective alternative for large body sections through water-bath curing.

Aspect	P35	P40	P45
Resin used	Polyester P35 (Biodur^®^)	Polyester P40 (Biodur^®^)	Polyester P45 (Hoffen^®^)
Typical slice thickness	4–8 mm	2–4 mm	2–3 mm (brain), large body sections
Curing process	UV + heat, dual curing	Simplified UV curing	Water bath at 40 °C
Main advantages	Excellent gray/white matter contrast in brain	High transparency, lower viscosity, more accessible, avoids UV using sunlight shadow	Avoids UV use, good clarity in large slices
Main disadvantages	Complex, bubble risk, requires thick safety glass	Sensitive to inadequate fixation	Less widespread, requires specific equipment
Common applications	Neuroanatomy, imaging correlation	Neuroanatomy, large body sections, medical and veterinary education	Large body sections, comparative research, veterinary anatomy

## Data Availability

The data presented in this study are available on request from the corresponding author.

## Supplementary Materials

The following supporting information can be downloaded at: https://www.mdpi.com/article/10.3390/polym17233177/s1, Table S1: Summary of AQUA domain risk assessments across the reviewed anatomical studies.
