# Supplementary material for: Polyester Sheet Plastination: Technical Foundations, Methodological Advances, Anatomical Applications, and AQUA-Based Quality Analysis
Source: Polymers (Basel). 2025 Nov 29;17(23):3177. doi: 10.3390/polym17233177 (PMC12694233; doi:10.3390/polym17233177)
Supplement: Supplementary file 1 [file polymers-17-03177-s001.zip › polymers-3879455-supplementary.pdf]

References	Domain 1: Objectives	Domain 2: Design	Domain 3: Methodology	Domain 4: Descriptive	Domain 5: Results report
von Hagens, G. (1987) [3]	Low	Low	Low	Low	Low
Weber & Henry (1992) [15]	Low	Unclear	Low	Low	Low
Weiglein (1993) [18]	Low	Unclear	Low	Low	Low
Weiglein (1996) [16]	Low	Unclear	Low	Low	Low
Barnett (1997) [24]	Low	Unclear	Low	Low	Unclear
Sora et al. (1999) [28]	Low	Low	Low	Low	Low
Sora & Brugger (2000) [26]	Low	Unclear	Low	Low	Low
Wadood et al. (2001) [14]	Low	Unclear	Low	Unclear	High
Latorre et al. (2003) [7]	Low	Low	Low	Low	Low
Barnett et al. (2005) [17]	Low	Low	Low	Low	Unclear
Genser-Strobl & Sora (2005) [33]	Low	Low	Low	Low	Low
Gao et al. (2006) [11]	Low	Unclear	Low	Low	Unclear
Henry & Latorre (2007) [5]	Low	Low	Low	Low	Low
Latorre & Henry (2007) [32]	Low	Low	Low	Low	Low
Sui & Henry (2015) [19]	Low	Low	Low	Low	Low
Weber et al. (2007) [6]	Low	Unclear	Low	Low	Unclear
Reed et al. (2008) [27]	Low	Low	Low	Unclear	Low
Üzel & Weiglein (2013) [25]	Low	High	Low	Unclear	Unclear
Zheng et al. (2014) [51]	Low	Low	Low	Low	Low
Chun et al. (2015) [41]	Low	Unclear	Unclear	Low	Unclear
Yuan et al. (2016) [48]	Low	Low	Low	Low	Low
Zhang et al. (2016) [50]	Low	Unclear	Low	Low	Unclear
Liu et al. (2017) [58]	Low	Low	Low	Low	Low
Zhang et al. (2017) [42]	Low	Low	Low	Low	Low
Zheng et al. (2017) [46]	Low	Low	Low	Low	Low
Liu et al. (2018) [57]	Low	Low	Low	Low	Low
Baptista et al. (2019) [8]	Low	Low	Low	Low	Low
Guerrero et al. (2019) [23]	Low	Low	Low	Low	Low
Okoye et al. (2019) [30]	Low	Unclear	Low	Low	Low
Okoye & Sui (2019) [29]	Low	Unclear	Low	High	Unclear
Ma et al. (2020) [40]	Low	Unclear	Low	Low	Unclear
Ottone et al. (2020) [9]	Low	Low	Low	Low	Low
Zheng et al. (2020) [54]	Low	Low	Low	Low	Low
Du et al. (2021) [45]	Low	Low	Low	Low	Low
Jiang et al. (2021) [34]	Low	Low	Low	Low	Low
Hwang et al. (2021) [44]	Low	Low	Low	Low	Low
Sun et al. (2021) [36]	Low	Low	Low	Low	Low
Zhang et al. (2021) [47]	Low	Low	Low	Low	Low

Chi et al. (2022) [49]	Low	Low	Low	Low	Low
Jiang et al. (2022) [35]	Low	Low	Low	Low	Low
Zhuang et al. (2022) [55]	Low	Unclear	Low	Low	Low
Zhang et al. (2023) [56]	Low	Low	Low	Low	Unclear
Jiang et al. (2023) [52]	Low	Low	Low	Low	Low
Li et al. (2023) [22]	Low	Unclear	Low	Low	Low
Zhang et al. (2023) [56]	Low	Low	Low	Low	Low
Cheng et al. (2025) [37]	Low	Unclear	Low	Low	Unclear
Shah et al. (2025) [38]	Low	Unclear	Low	Low	Low
Zhang et al. (2025a) [43]	Low	Low	Low	Low	Low
Zhang et al. (2025b) [53]	Low	Unclear	Low	Low	Unclear

Supplementary Table S1. Summary of AQUA domain risk assessments across the reviewed anatomical studies. Each domain was evaluated for risk of bias according to the AQUA tool: Domain 1 (Objectives), Domain 2 (Study Design), Domain 3 (Methodology), Domain 4 (Descriptive Anatomy), and Domain 5 (Results Reporting). Risk levels are classified as: Low (green), Unclear (yellow), and High (red), reflecting the methodological quality of each article.
